# Crack mechanism and experimental verification on straightening of AZ31B magnesium alloy plate

**DOI:** 10.1038/s41598-023-36396-7

**Published:** 2023-06-05

**Authors:** Rong-Jun Wang, Qi Zhou, Xiao-Zhong Du, Yu-Shan Li, Peng-Chong Zhang, Guang-Feng Li, Zhi-Quan Huang, Li-Dong Ma, Lian-Yun Jiang

**Affiliations:** 1 School of Mechanical Engineering, Taiyuan University of Science and Technology, Taiyuan, 030024 China; 2 Upgrading Office of Modern College of Humanities and Sciences of Shanxi Normal University, Linfen, 041000 China

**Keywords:** Mechanical engineering, Metals and alloys

## Abstract

When plates with edge cracks in the rolling process is straightened by cyclic tensile and compressive stress, the tip of edge crack always accompanied by stress concentration, which leads to crack propagation. In this paper, damage parameters are imported into the plate straightening model based on determining the GTN damage parameters of magnesium alloy materials by inverse finite element calibration method, the influence of different straightening process schemes and prefabricated V-shaped crack geometry on crack growth is analyzed through the way of the combination of simulation and straightening experiment. The results show that the peak values of equivalent stress and equivalent strain under each straightening roll appear at the crack tip. The value of longitudinal stress and equivalent stain decrease with the distance to crack tip becomes larger. The peak value of longitudinal stress appears when the crack circumferential angle is about 100°, and the crack tip is easy to form crack propagation; when the plate passes roll 2 and roll 4, the equivalent stress and strain concentration at the crack tip are most obvious; when the reduction reaches a certain degree, the void volume fraction (VVF) reaches the VVF of the material breaking; with the increase of the entrance reduction, the number of VVF at the crack tip which reaches the material fracture increases, and the length of crack propagation increases; the stress concentration at the tip of V-shaped crack with large length–width ratio is obvious, and the VVF is more likely to reach the VVF at the time of material fracture, crack initiates and propagates easily.

## Introduction

Rolling process has become one of the main methods of magnesium (Mg) alloy plate production, it has the characteristics of refining grain, improving structure, and significantly improving the mechanical properties of the alloy^1^. However, Mg has a special hexagonal close-packed (HCP) structure, with few slip systems at room temperature, poor performance in pressure processing and plastic processing, and edge cracks will appear during rolling. On the other hand, the rolled Mg alloy plates will also have defects such as buckling and wave during transportation. Straightening, as an important link in the processing and forming process of Mg alloy plates, aims to improve the flatness and reduce residual stress effectively^2^. When Mg alloy plates with the above defects are straightened by cyclic tensile and compressive stress, stress concentration is easy to occur at the edge cracks, causing the initiation and propagation of micro cracks at the crack tip, and even causing the plates to fracture during the straightening process.

Domestic and foreign scholars have conducted a detailed study on the straightening of steel^3–5^, and its straightening theory can be used for straightening Mg alloy plates. Similarly, many scholars have studied the initiation and propagation of cracks. Paris et al.^6^ characterized the crack propagation by J-integral. JR resistance curve can be used to analyze the steady-state crack propagation when studying the crack propagation of elastic–plastic materials. Gong et al.^7^ reached the same conclusion as PC PARIS when studying the unstable crack propagation in circumferentially notched short cylinders and found that when the crack propagates from initiation to destabilization, the grid at the crack tip deforms obviously due to the large stress concentration. With the increase of stress, the grid distortion gradually occurs and becomes more and more obvious with the increase of stress. Yu et al.^8^ simulated the rolled piece with corner transverse crack and longitudinal crack by using plain-barreled vertical roll and shape vertical roll through finite element. The crack propagation and closure were observed by pre-fabricating V-shaped crack at the corner of the plate. The research^9^ shows that the stress concentration will occur in the Mg alloy with low temperature level, which will cause edge cracks of the Mg alloy. Zeng et al.^10^ found the crack propagation of Mg alloy is a relatively complex process through molecular dynamics simulation research. Due to the external tensile stress, the relatively weak atomic bond at the crack tip of Mg alloy breaks and the crack begins to destabilize and propagate. The crack initiation of Mg alloy will be accompanied by dislocation emission of slip system, which will eventually lead to complete failure of alloy. Zhang et al.^11^ investigated temperature changes, edge cracks and rolling force during rolling of magnesium alloy sheet. They found that hole development, shear deformation and accumulative plastic strain cause the plastic-damage in Mg sheet. Liu et al.^12^ designed Mg alloy plate specimens with prefabricated cracks, and studied the evolution rules during rolling process, which provided basis for restraining edge crack initiation and propagation of Mg alloy strip during production. Huang et al.^13^ investigated the influence of prefabricated crown on edge cracking of rolled AZ31 Mg alloy plate. They found that the crack generation can be effectively controlled by using prefabricated crown rolling process. Jia et al.^14^ studied the deformation and fracture behaviors of as-cast AZ31B Mg alloy by uniaxial compression experiments and finite element simulation with wide ranges of temperature and strain rate, they found that under low temperature and high strain rates, the observed sharp in stress caused by micro-cracks initiation and propagation can be readily produced. Jia et al.^15^ quantitatively analyze the damage distributions induced by rolling deformation in transverse direction (TD) for twin-roll casted (TRCed) AZ31 Mg alloy and found that in the longitudinal section of rolled sheets, the edge crack is dominated by 45° as the temperature decreases and the reduction rate increases, the cracking deep along the TD increases.

In summary, there are relatively many studies on rolling cracks, little research on edge cracking mechanism of Mg alloy plate with original edge cracks in straightening process. In this paper, the mechanism of initiation, propagation, and evolution of edge crack during straightening process of Mg alloy plate is studied taking AZ31B Mg alloy plate with original edge crack as the research object.

## GTN damage model and determination of damage parameters

### Maximum tensile stress theory

From the point of view of macro-scale mechanics, according to mechanics of materials and fracture mechanics, there are many methods can be applied to study the propagation of mixed mode crack, such as the energy release rate theory, the maximum tensile stress theory (maximum circumferential stress theory) and the strain energy density factor theory, among which the maximum tensile stress theory is most used in engineering practice. Erdogan et al.^16^ consider that the main factor of crack propagation is the maximum tensile stress at the crack tip. The crack tip splits when the circumferential tensile stress reaches the critical value of crack initiation.

The maximum tensile stress theory proposed in fracture mechanics has the following basic assumptions^17^:

1. The maximum stress exists at the circumferential position of the crack tip, and crack propagates along the direction of maximum stress.
2. The crack propagates when the maximum circumferential stress increases to the critical value for crack cracking.

Based on the above two assumptions, the crack angle and the critical point of crack cracking can be known. According to the superposition principle, the stress field around the top of I-II composite crack under polar coordinates can be derived.

1 $$ {\text{ }}\left. {\begin{array}{*{20}c}    {\sigma _{r}  = \frac{1}{{2\sqrt {2\pi r} }}\left( {K_{I} (3 - \cos \theta )\cos \frac{\theta }{2} + K_{{II}} (3\cos \theta  - 1)\sin \frac{\theta }{2}} \right)}  \\    {\sigma _{\theta }  = \frac{1}{{2\sqrt {2\pi r} }}\cos \frac{\theta }{2}\left( {K_{I} \cos ^{2} \frac{\theta }{2} - \frac{3}{2}K_{{II}} \sin \theta } \right)}  \\    {\tau _{{r\theta }}  = \frac{1}{{2\sqrt {2\pi r} }}\cos \frac{\theta }{2}\left( {K_{I} \sin \theta  + K_{{II}} (3\cos \theta  - 1)} \right)}  \\   \end{array} } \right\} $$

where *σ*_*r*_ denotes the radial stresss, *σ*_*θ*_ is the circumferential stress, *τ*_*rθ*_ denotes the shear stress, *θ* is the angle of cracking, *r* and *θ* are the coordinates under polar coordinates, $K_{I} = \sigma \sqrt {\pi a}$, which is the stress intensity factor of mode-I crack,$K_{II} = \tau \sqrt {\pi a}$ which is the stress intensity factor of mode-II crack, a denotes half of the crack length.

**Figure 1:**
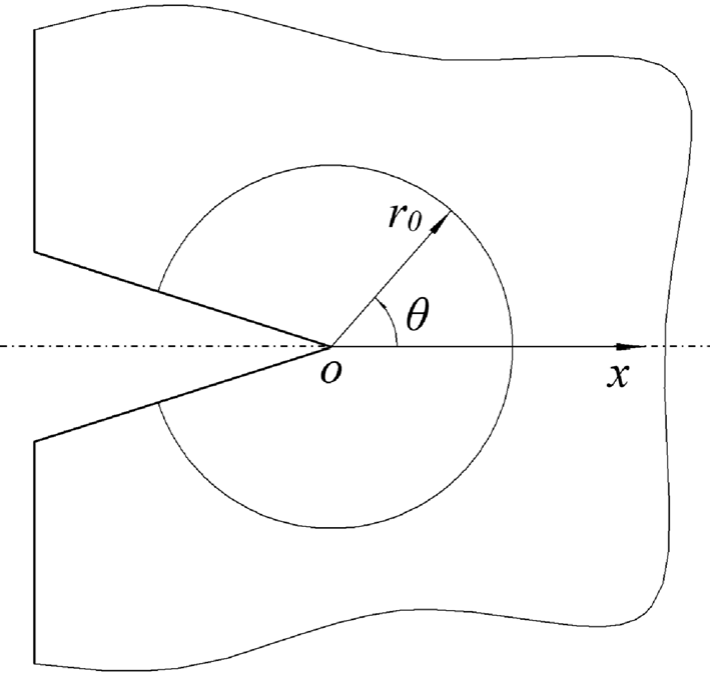
Stress field at crack tip.

When *r* → 0, the components of stress in all directions are close to infinity at the crack tip, so take a smaller distance *r* = *r*_0_ at the crack tip, we can know the circumferential stress *σ*_*θ*_ (Fig. 1) at different points on the circumference, the crack angle *θ*_0_ at the crack tip can be obtained by circumferential stress.

The second equation in Eq. (1) is derived according to the extreme value condition, if *θ* = *θ*_0_, the extreme value condition of circumferential stress can be met, namely

2 $$ \cos \frac{{\theta_{0} }}{2}(K_{I} sin\theta_{0} + K_{II} (3cos\theta_{0} - 1)) = 0 $$

When *θ*_0_ =  ± *π*, Eq. (2) is met, but this value has no real meaning, so *θ*_0_ depends on:

3 $$ K_{I} sin\theta_{0} + K_{II} (3cos\theta_{0} - 1) = 0 $$

Get the fracture angle *θ*_0_ according to Eq. (3), substitute into the second equation in Eq. (1), then the equation of the maximum circumferential stress on the circumference is:

4 $$ (\sigma_{\theta } )_{\max } = \frac{1}{{\sqrt {2\pi r_{0} } }}\cos \frac{{\theta_{0} }}{2}\left( {K_{I} \cos^{2} \frac{{\theta_{0} }}{2} - \frac{3}{2}K_{II} \sin \theta_{0} } \right) $$

According to hypothesis (2), the corresponding cracking discriminant is obtained:

5 $$ (\sigma_{\theta } )_{\max } = (\sigma_{\theta } )_{c} $$

where (*σ*_*θ*_)_*c*_ is the critical stress value of the maximum circumferential stress at the crack tip, which is obtained according to the critical stress intensity factors *K*_*IC*_ of model I crack.

Since the model I crack always propagates along the initial crack surface, *θ*_*0*_ = 0. Substitute *K*_*II*_ = 0, *θ*_*0*_ = 0, *K*_*I*_ = *K*_*IC*_ into Eq. (4), we can get:

6 $$ (\sigma_{\theta } )_{c} = \frac{{K_{IC} }}{{\sqrt {2\pi r_{0} } }} $$

Substitute Eqs. (4) and (6) into Eq. (5), as follows:

7 $$ \cos \frac{{\theta_{0} }}{2}\left( {K_{I} \cos^{2} \frac{{\theta_{0} }}{2} - \frac{3}{2}K_{II} \sin \theta_{0} } \right) = K_{IC} $$

The above criterion is based on the maximum tensile stress theory in fracture mechanics. It can be used as cracking criterion for I–II mixed mode crack.

### GTN damage model

GTN damage model revised by Tvergaard and Needleman et al.^18,19^ has been wildly used to describe and predict the micro porous damage fracture of ductile metals. The model is expressed as follows:

8 $$ \Phi = \left( {\frac{{\sigma_{eq} }}{{\sigma_{m} }}} \right)^{2} + 2f^{*} q_{1} \cosh \left( {\frac{{3q_{2} \sigma_{H} }}{{2\sigma_{m} }}} \right) - 1 - q_{3} (f^{*} )^{2} = 0 $$

where Φ denotes the yield surface, *σ*_*eq*_ is the macro-scale equivalent Mises stress, *σ*_*m*_ is the flow stress of the basis material, *σ*_*H*_ denotes the hydrostatic state of stress, $f^{*}$ is the equivalent VVF, *q*_1_, *q*_2_, *q*_3_ are three modified damage parameters, their values are different for different metal. Koplik et al.^20^ founds that *q*_1_, *q*_2_, *q*_3_ are determined by the yield stress and plasticity index of the material.

The effective volume fraction $f^{*}$ is a function of *f* which denotes the VVF of the material, the function is:

9 $$ f^{*} = \left\{ \begin{gathered} f\quad \quad \quad \quad \quad \quad \;f \le f_{c} \hfill \\ f_{c} + k(f - f_{c} )\;\;f > f_{c} \hfill \\ \end{gathered} \right. $$

10 $$ k = \frac{{\frac{1}{{q_{1} }} - f_{c} }}{{f_{F} - f_{c} }} $$

where *f*_*F*_ denotes the VVF when the material breaks, *f*_*c*_ is the critical VVF; *k* is the void growth factor.

The damage evolution of material is mainly due to the change of VVF caused by the growth of original voids and the nucleation of new voids. The overall damage evolution is expressed as:

11 $$ \dot{f} = (\dot{f})_{g} + (\dot{f})_{n} $$

where $(\dot{f})_{g}$ denotes the growth change of the original void, $(\dot{f})_{n}$ is the nucleation change of the new void.

Because the basis material cannot be compressed, the growth of the original void is only related to the macro-scale plastic volume deformation inside the material, namely:

12 $$ (\dot{f})_{g} = (1 - f)\dot{E}_{kk}^{p} $$

$\dot{E}^{p}_{kk}$ is three times the macro-scale plastic volumetric strain rate.

The growth of new void will cause an increase in the volume fraction of voids in the material, namely:

13 $$ (\dot{f})_{n} = A\dot{\varepsilon }_{e}^{P} $$

The researchers consider that for the nucleation of material micropores, the percentage of void volume increases due to the formation of new voids, if only consider the nucleation mechanism of plastic deformation, it is believed that under the condition of compressive stress, new micropores will not appear, and the effect of tensile stress will be formed, namely:

14 $$ A = \left\{ \begin{gathered} \frac{{f_{n} }}{{hS_{N} \sqrt {2\pi } }}\exp \left( { - \frac{1}{2}\left( {\frac{{\varepsilon_{e}^{p} - \varepsilon_{N} }}{{S_{N} }}} \right)^{2} } \right)\quad \sigma_{m} \ge 0 \hfill \\ 0\quad \quad \quad \quad \quad \quad \quad \quad \quad \quad \quad \quad \quad \quad \quad \,\sigma_{m} < \, 0 \hfill \\ \end{gathered} \right. $$

*A* denotes void nucleation coefficient, *f*_*n*_ is void nucleation parameter of metal material, *h* is hardening modulus of material, $\varepsilon_{e}^{p}$ denotes equivalent plastic strain of material, *S*_*N*_ denotes standard deviation of void nucleation strain, *ε*_*N*_ is the average strain of void nucleation in metallic material.

In the material, when the VVF *f* increases to the critical VVF *f*_*c*_, void polymerization occurs. When *f* increases to the fracture VVF *f*_*F*_, macro-scale cracks occur.

To sum up, the determination of GTN damage model parameters in materials requires not only the stress–strain relationship, but also the *ε*_*N*_, *S*_*N*_, *f*_*n*_, *f*_0_ (initial void volume ratio), *f*_*c*_, *f*_*F*_ and other parameters.

### Determination of damage parameters

GTN damage model parameters include *q*_1_, *q*_2_, *q*_3_, *f*_0_, *f*_*c*_, *f*_*F*_, *f*_*N*_, *S*_*N*_, *ε*_*N*_, these parameters can effectively predict the damage and fracture in the material forming process. The most common method to determine material damage parameters is inverse finite element method.

According to Tvergaard’s suggestion^18^, the damage parameters of the interaction between voids of commonly used metal materials, which are respectively *q*_1_ = 1.5, *q*_2_ = 1.0 and *q*_3_ = 2.25. Compared with precious numerical simulations and experiments, the values of these three damage parameters are applicable to most metal materials, which have also been confirmed by many scholars at home and abroad. Wang^21^ confirmed the VVF of Mg alloy during fracture by debugging the simulation model for many times. The parameters are as follows, *f*_*F*_ = 0.1279, *f*_*c*_ = 0.073, *f*_0_ = 0.001. Through literature review^22^, the value range of *ε*_*N*_ and *f*_*N*_ are respectively 0.15–0.3 and 0.01–0.07, generally, there is no fixed value. For typical metals, the value range of *S*_*N*_ is 0.05–0.1.

In this paper, AZ31B Mg alloy plate is taken as the research object, and its corresponding chemical composition is shown in Table 1. The plate is stretched with a universal tensile testing machine. The material parameters of AZ31B Mg alloy shown in Table 2 are obtained through tensile test, and the engineering stress and engineering strain are transformed into real stress and real strain parameters. The fundamental performance parameter of AZ31B Mg alloy obtained from the tensile test are imported into the material properties of finite element software ABAQUS^23^, and then the boundary conditions are established in the tensile finite element model according to the actual tensile test conditions for loading, so that the boundary conditions of tensile simulation are consistent with the real experimental boundary conditions.

**Table 1:** Chemical composition (wt%) of AZ31B Mg alloy.

Model	Mg	Al	Si	Ca	Zn	Mn	Fe	Cu	Ni
AZ31B	Allowance	3.13	0.0218	0.04	1.030	0.36	0.001	0.0003	0.0004

**Table 2:** Basic parameters of AZ31B magnesium alloy sheet material.

Parameter	Value
Young’s modulus	44,800 MPa
Poisson’s ratio	0.35
Density	1.78 g/cm^3^
Yield strength	200 MPa

**Figure 2:**
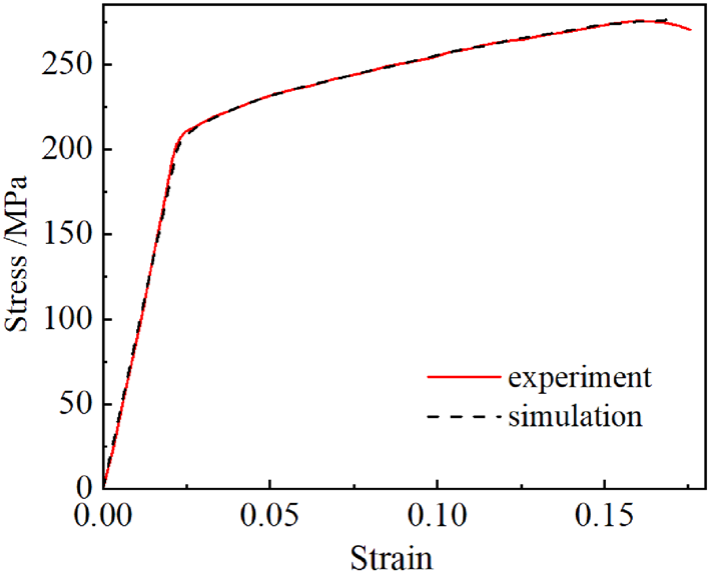
Comparison of experimental and simulated true stress–strain curves.

In ABAQUS, the tensile simulation test piece is meshed, and the middle part of the tensile test piece is refined by local mesh, using reduced integration, C3D8R eight-node linear hexahedron element, and hourglass control^23^. By continuously selecting and adjusting the corresponding GTN damage parameters, the experimental results and simulation results are plotted as shown in Fig. 2, and a group of curves with small coincidence error are selected.

It is found that reasonable GTN material damage model parameters can better simulate the results of uniaxial tension by comparing several groups of stress–strain fitting curve of uniaxial tensile tests and numerical simulation. The damage parameter values of the GTN damage model of AZ31B Mg alloy material obtained by the inverse finite element method are shown in Table 3.

**Table 3:** GTN model damage parameters of AZ31B Mg alloy.

*q*_1_	*q*_2_	*q*_3_	*f*_0_	*f*_*c*_	*f*_*F*_	*S*_*N*_	*ε*_*N*_	*f*_*N*_
1.5	1	2.25	0.001	0.073	0.1279	0.05	0.2	0.04

### Ethical approval

The article follows the guidelines of the Committee on Publication Ethics (COPE) and involves no studies on human or animal subjects.

## Simulation and experiment

### Finite element model

**Figure 3:**
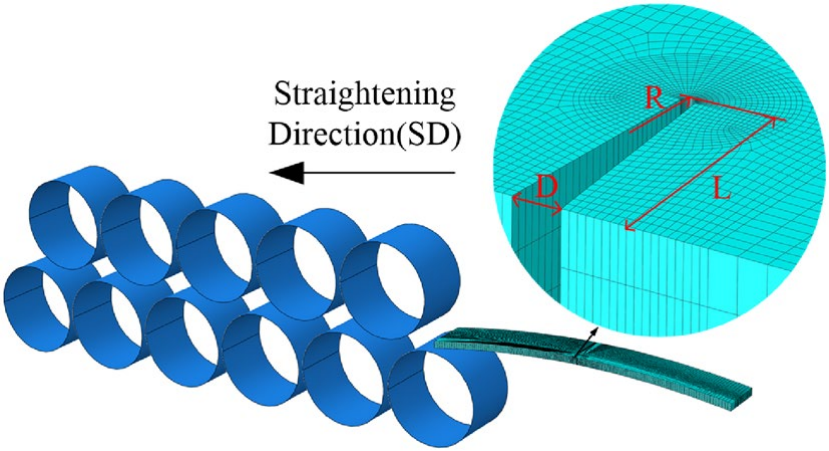
Straightening model of Mg alloy plate with prefabricated V-shaped edge crack.

As shown in Fig. 3, the finite element model of the straightening process of AZ31B Mg alloy plate at room temperature is established with the finite element software ABAQUS. The straightening rolls are named roll 1 to roll 11 from the inlet to the outlet, the basic parameters are shown in Table 4. The process schemes of straightening process are shown in Table 5.

**Table 4:** Basic parameters (mm).

Parameter	Value
Roller diameter	95
Roller spacing	100
Plate length	400
Plate width	100
Plate thickness	6.8
Wave height	18

**Table 5:** Test straightening process schemes.

Process scheme	Reduction of entrance (mm)	Reduction of exit (mm)	Straightening speed (mm/s)
1	1.5	1	190
2	2.2	1	190
3	3	1	190
4	3.5	1	190

The GTN damage parameters of AZ31B Mg alloy determined by tensile test as shown in Table 3 are imported into the material properties of the plate. The element type is eight-node linear hexahedral element C3D8R, which adopts reduced integration and hourglass control^23^. At the same time, V-shaped edge cracks are prefabricated on one side of AZ31B Mg alloy plate in the model. The size and grid division of V-shaped cracks are shown in Fig. 3. The width D is 1 mm, the length L is 1 mm, 3 mm, and 5 mm respectively, the radius R at the crack tip is 0.15 mm, and the gap between the V-shaped cracks is about 20 mm. The damage state variable is imported into the model to control the element failure. When the VVF reaches the damage parameter set in the material model, the elastic modulus of the element decreases until the bearing capacity is lost. The crack initiation and propagation are simulated by the automatic deletion of the element.

### Straightening test

The straightening machine used in the experiment is the eleven rollers’ full hydraulic straightening machine of Taiyuan University of Science and Technology. The wire cutting machine is used to prefabricate the V-shaped cracks at the edge of the plate. The shape, size and gap are the same as those of the finite element model.

## Result

### Stress and strain analysis of the front edge of crack in straightening process

**Figure 4:**
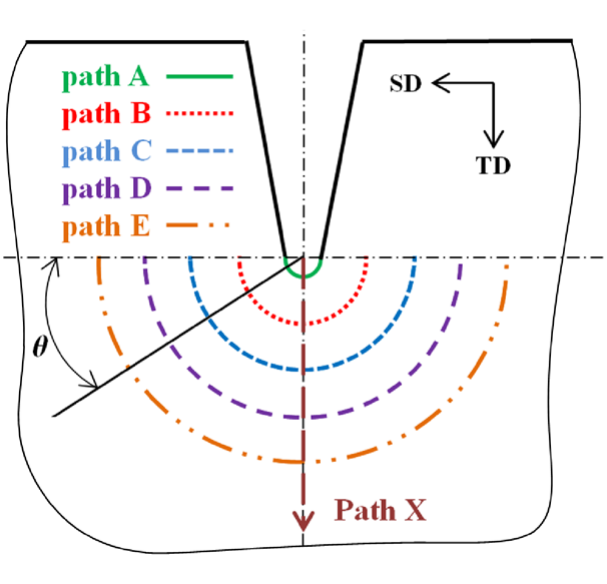
Path at the crack tip.

**Figure 5:**
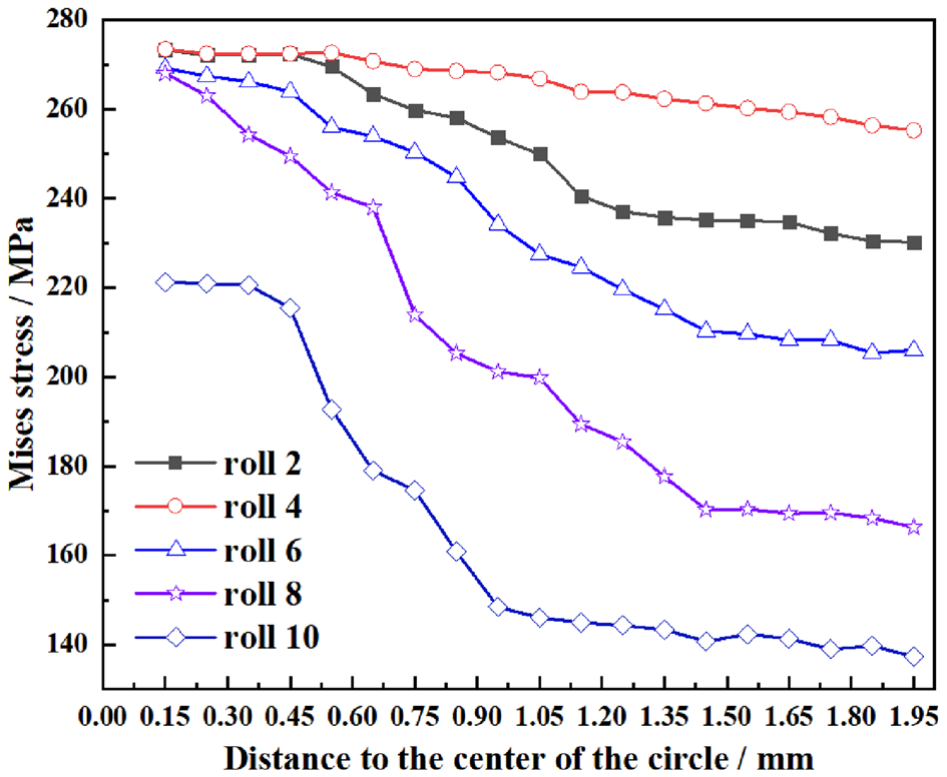
Mises stress of direction X under different straightening rollers.

**Figure 6:**
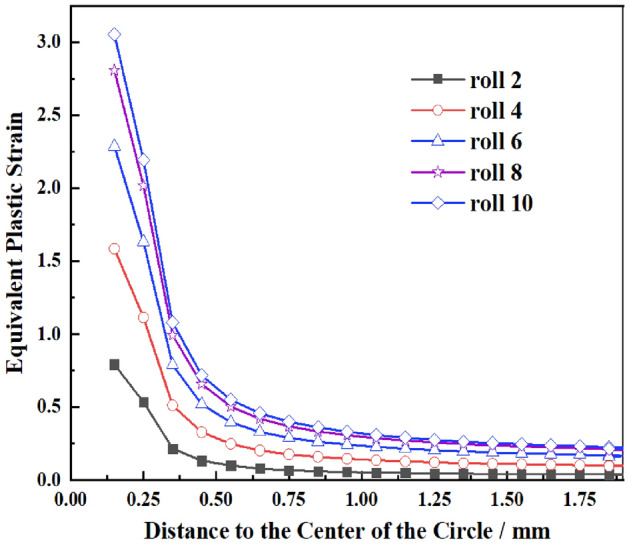
Equivalent plastic strain of direction X under different straightening rollers.

To quantitatively analyze the changes of Mises stress at different distances at the crack tip, path X starting from the center of the prefabricated crack circle and perpendicular to the straightening direction (SD) as shown in Fig. 4 is established along the middle of the crack tip with length of 1.95 mm. When the plate is straightening with process scheme 2 shown in Table 5, the relevant data of the operation file is extracted when the prefabricated crack (L = 5 mm) is located below the upper row of rolls, and the stress diagram of path X under different straightening rolls is drawn as shown in Fig. 5, and the equivalent plastic strain diagram is shown in Fig. 6. It can be seen from Fig. 5 that the equivalent stress at the tip of the prefabricated crack is the largest. With the increase of the distance from the crack tip, the equivalent stress gradually decreases. It can be inferred that the crack tip is most likely to cause crack initiation and propagation. At the same time, the equivalent stress concentration of the prefabricated crack is the most obvious when passing through roll 2 and roll 4, and the stress is small when passing through roll 10. It can be inferred that the prefabricated crack is most prone to initiate and propagate when passing through roll 2 and roll 4.

It can be seen from Fig. 6 that the equivalent plastic strain at the crack tip is always the largest. The farther away from the crack tip, the smaller the equivalent plastic strain. The equivalent plastic value gradually increases as the straightening proceeds until the front edge of the crack tip is separated from the straightening roll, and the equivalent plastic strain no longer changes. It can also be inferred that the crack at the prefabricated crack tip is easy to initiate and propagate.

To further study the distribution law of the stress and strain at the crack tip during the straightening process, the stress and strain values on the arc path at a certain distance from the crack tip are analyzed to study the cracking of the crack at the crack front edge. As shown in Fig. 4, take semicircle paths A, B, C, D, E with the same center as the crack tip (radius of 0.15 mm) on the lower surface of the plate and radius of 0.15 mm, 0.55 mm, 0.95 mm, 1.35 mm, 1.75 mm respectively. And define the circumference angle *θ* which refers to the included angle between SD and the line between the point on the arc and the center of the circle.

**Figure 7:**
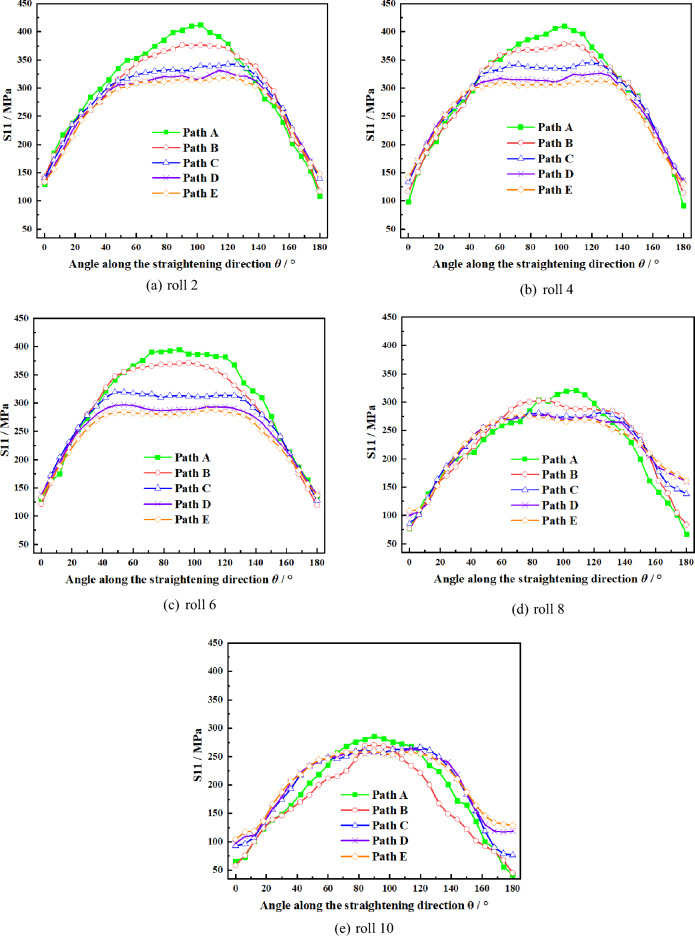
Stress distribution of S11 on different paths of crack on plate bottom surface.

Because the crack propagation is caused by the tensile stress at the crack tip, it is of great significance to analyze the stress distribution at the front edge of the crack when the plate is tensioned during the straightening process. When the plate is straightening with process scheme 2 shown in Table 5, as the edge prefabricated crack (L = 5 mm) is located under the upper row of rolls, the change of the longitudinal stress component S11 of the path A-E at the front edge of crack on the tensile side of the plate is shown in Fig. 7.

According to Fig. 7, the longitudinal stress value of path A at the point of crack tip on the tension side of the plate under the upper straightening rollers is always the maximum, the farther away from the crack tip, the longitudinal stress gradually decreases, and the peak value appears when *θ* = 100°, which indicates that when straightening Mg alloy plate, fracture failure is easy to occur at the position about 100 degrees between the plate and the straightening direction due to the influence of contact angle between plate and straightening roll during straightening process.

### Effect of reduction on crack growth

**Figure 8:**
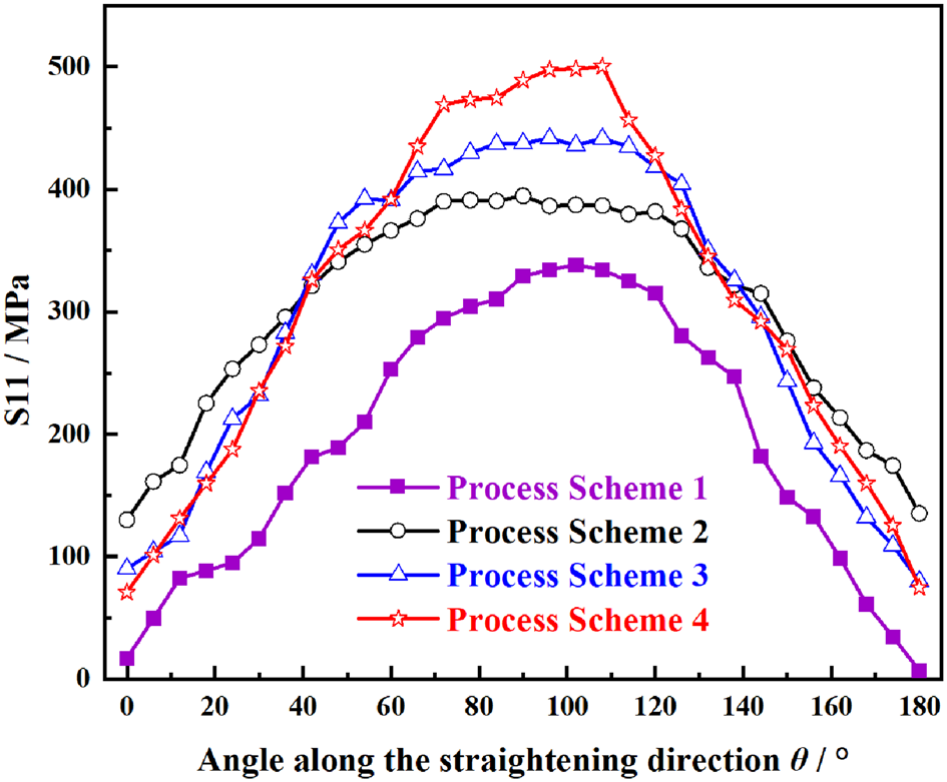
Stress component S11 at crack tip under roll 2 in different process schemes.

**Figure 9:**
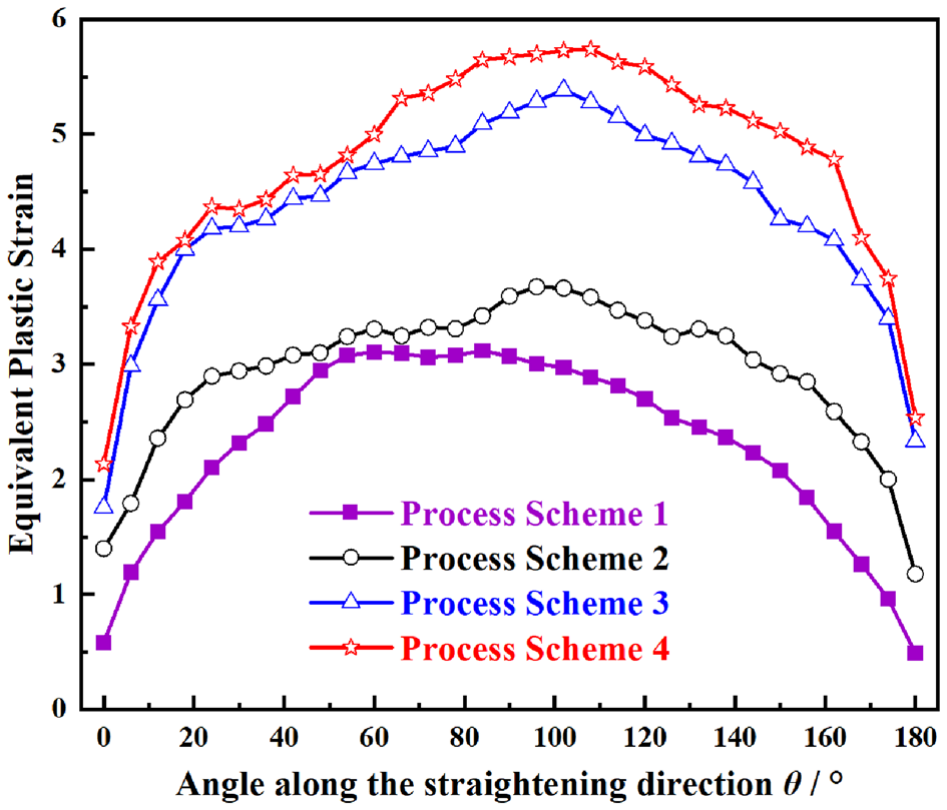
Equivalent plastic strain of crack tip leading edge under different reduction.

**Figure 10:**
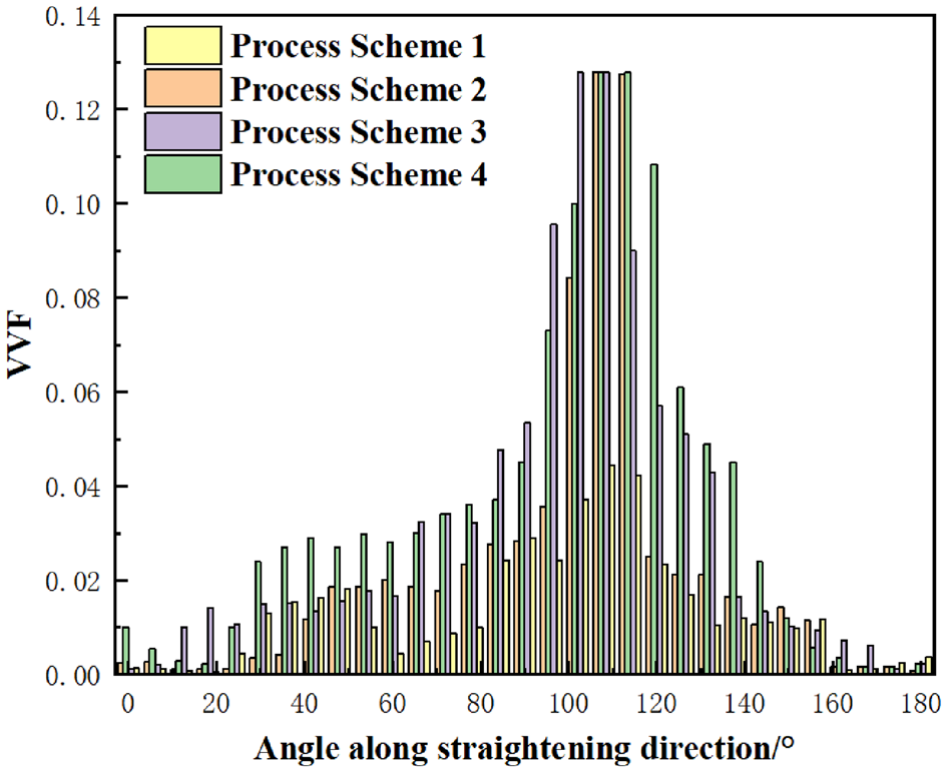
VVF of holes at the front edge of V-crack tip.

To analyze the influence of reduction on cracking at the crack tip, the distribution laws of equivalent stress, equivalent plastic strain and VVF of path A as shown in Fig. 4 for prefabricated cracks (L = 5 mm) are analyzed, as shown in Figs. 8, 9, and 10, respectively.

From the previous analysis, the longitudinal stress S11 at the front edge of crack is one of the important factors causing the growth of edge cracks. Due to the inclined distribution of the upper straightening row rolls, when the edge cracks of Mg alloy plate are roll 2, the reduction is greater than that of the rear rolls. Therefore, it is of great significance to study the stress component S11 of plates passing roll 2 with different process schemes. The corresponding S11 stress at the crack tip passing roll 2 under different process schemes is shown in Fig. 8. The longitudinal stress component S11 increases with the increase of reduction, and the crack tip initiates and propagates more easily.

The equivalent plastic strain at the front edge of the crack tip under different reduction is shown in Fig. 9. When the angle between the front edge of the crack tip and the SD is about 100°, the equivalent plastic strain reaches the peak value and the plastic strain around the crack tip decreases gradually. The equivalent plastic strain of the prefabricated crack tip increases with the increase of the reduction, and obvious plastic deformation occurs at the front edge of the crack tip. The maximum equivalent strain plastic strain always appears at the front edge of the crack tip, and approaches 6 when the reduction is 3.5 mm. The front edge of the crack tip is the most fragile part of the plate, which is consistent with the stress analysis results.

When the VVF value in the simulation reaches the failure threshold *f*_*F*_ which is 0.1279, the element of the V-shaped crack tip failure and deleted to determine that the crack occurs, and a small crack initiate. After simulation of the process schemes shown in Table 5, VVF of holes at the front edge of V-shaped crack tip is shown in Fig. 10.

**Figure 11:**
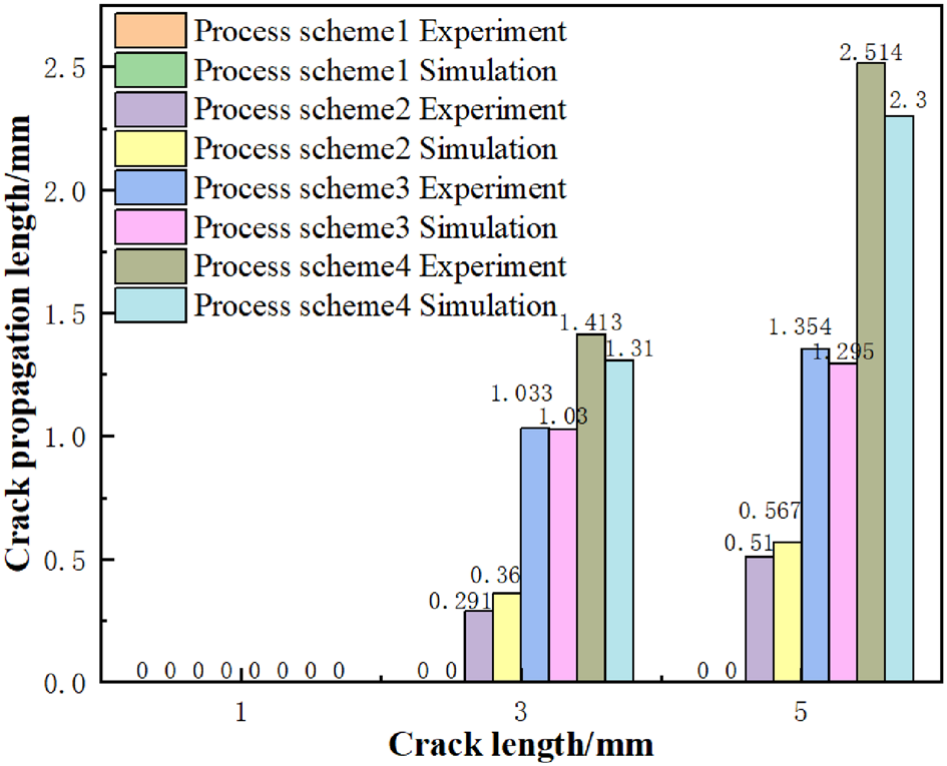
Effect of reduction on crack propagation length.

Under different process schemes, the original picture of crack and results of crack propagation obtained from experiment and simulation are listed in Tables 6, 7, and 8. The variation rule of reduction with crack growth length obtained by simulation and experiment is shown in Fig. 11. When the plate is straightened with process scheme 1 (with reduction of entrance is 1.5 mm), there is no crack initiation and propagation in all prefabricated edge cracks. Crack growth phenomena of different lengths are found in process scheme 2, 3 and 4 (with reduction of entrance is 2.2 mm, 3 mm, and 3.5 mm respectively), and the crack growth length increases with the reduction of entrance. Because the V-shaped crack produce larger additional tensile stress with the increase of the reduction, the stress concentration at the crack tip becomes more obvious, the deformation of the crack tip is intensified, the equivalent plastic strain increases, the voids inside the material coalesce, finally results in the increase of the VVF. When the reduction reaches a certain level and the VVF reaches *f*_*F*_, the V-shaped crack begins to crack at the tip, small cracks initiate. With the increase of the reduction, the number of times that the VVF of a single V-shaped crack reaches *f*_*F*_, the length of crack growth increases; this is also confirmed by the fact that the finite element fits the experimental results well.

**Table 6:** Crack growth length of notches with different aspect ratio in process scheme 2.

	Result of experiment	Result of simulation
D = 1 mm L = 3 mm		
D = 1 mm L = 5 mm

**Table 7:** Crack growth length of notches with different aspect ratio in process scheme 3.

	Result of experiment	Result of simulation
D = 1 mm L = 3 mm		
D = 1 mm L = 5 mm

**Table 8:** Crack propagation length of notches with different aspect ratio in process scheme 4.

	Result of experiment	Result of simulation
D = 1 mm L = 3 mm		
D = 1 mm L = 5 mm

### Effect of crack size on crack propagation

Figure 11 shows the crack growth of V-shaped cracks with different lengths after straightening under four process schemes. When the length of the prefabricated crack is 1 mm, there is no crack initiation at the tip of the V-shaped crack after straightening in the four process schemes. This is because the length–width ratio of the crack is small and the stress concentration at the tip of the crack is not obvious, the VVF does not reach *f*_*F*_, so no element will be deleted due to failure. When the length of prefabricated crack is 5 mm, the length–width ratio is large, the local stress concentration at the tip of the crack is obvious, and the equivalent plastic strain is larger, which can promote the increase of the VVF, make it reach *f*_*F*_ easily, causing crack initiation and propagation. Therefore, both the simulation and experimental date in Fig. 10 show that the more obvious the stress concentration at the tip of the prefabricated crack is, the easier it is for the VVF at the tip of the crack to reach *f*_*F*_, and the crack initiate and propagate easily.

## Conclusion

In this paper, the tensile test and simulation of Mg alloy plates are carried out. Based on determining the GTN damage parameters of AZ31B Mg alloy with reverse finite element method, the damage parameters are imported into the straightening model for simulation and experiment. The results show that:

1. The GTN damage parameters which are consistent with AZ31B Mg alloy used in experiment are determined.
2. The peak value of equivalent stress and equivalent strain under each straightening roll appears at the tip of the crack. The value of longitudinal stress and equivalent stain decrease with the distance to crack tip becomes larger. The peak value of longitudinal stress appears when the crack circumferential angle is about 100°, and the crack tip is easy to propagate. When the plate passes roll 2 and roll 4, the equivalent stress and strain concentration at the crack tip are most obvious.
3. When the reduction of entrance reaches a certain level, the VVF reaches *f*_*F*_ (the VVF when the material breaks.) With the increase of reduction of entrance, the stress and equivalent plastic strain increase, and the number of times that VVF reaches *f*_*F*_ increases, and the length of crack growth increases.
4. The stress concentration at the tip of V-shaped crack with large length–width ratio is obvious, and the VVF is more likely to reach *f*_*F*_, the crack initiate and propagate easily.

## Data Availability

The datasets supporting the results of this article are included within the article.
